# On the Microstructure of Laser Beam Powder Bed Fusion Alloy 718 and Its Influence on the Low Cycle Fatigue Behaviour

**DOI:** 10.3390/ma13225198

**Published:** 2020-11-17

**Authors:** Arun Ramanathan Balachandramurthi, Nitesh Raj Jaladurgam, Chamara Kumara, Thomas Hansson, Johan Moverare, Johannes Gårdstam, Robert Pederson

**Affiliations:** 1 Department of Engineering Science, University West, SE-461 86 Trollhättan, Sweden; chamara.kumara@hv.se (C.K.); thomas.hansson@hv.se (T.H.); johan.moverare@liu.se (J.M.); robert.pederson@hv.se (R.P.); 2 Department of Physics, Chalmers University of Technology, SE-412 96 Gothenburg, Sweden; niteshj@chalmers.se; 3 GKN Aerospace Sweden AB, SE-461 38 Trollhättan, Sweden; 4 Department of Management and Engineering, Linköping University, SE 581 83 Linköping, Sweden; 5 Qunitus Technologies AB, SE-721 66 Västerås, Sweden; johannes.gardstam@quintusteam.com

**Keywords:** powder bed fusion, additive manufacturing, fatigue, hot isostatic pressing, superalloys

## Abstract

Additive manufacturing of Alloy 718 has become a popular subject of research in recent years. Understanding the process-microstructure-property relationship of additively manufactured Alloy 718 is crucial for maturing the technology to manufacture critical components. Fatigue behaviour is a key mechanical property that is required in applications such as gas turbines. Therefore, in the present work, low cycle fatigue behaviour of Alloy 718 manufactured by laser beam powder bed fusion process has been investigated. The material was tested in as-built condition as well as after two different thermal post-treatments. Three orientations with respect to the building direction were tested to evaluate the anisotropy. Testing was performed at room temperature under controlled amplitudes of strain. It was found that defects, inclusions, strengthening precipitates, and Young’s modulus influence the fatigue behaviour under strain-controlled conditions. The strengthening precipitates affected the deformation mechanism as well as the cycle-dependent hardening/softening behaviour. The defects and the inclusions had a detrimental effect on fatigue life. The presence of Laves phase in LB-PBF Alloy 718 did not have a detrimental effect on fatigue life. Young’s modulus was anisotropic and it contributed to the anisotropy in strain-life relationship. Pseudo-elastic stress vs. fatigue life approach could be used to handle the modulus-induced anisotropy in the strain-life relationship.

## 1. Introduction

Additive manufacturing (AM) has had a disruptive influence in the manufacturing sector in the last decade and has become a core technology of the fourth industrial revolution, *Industry 4.0*. Metal AM technology is maturing and evolving at a dramatic rate but at the current level of technology development, it is better suited for low-volume-sector due to the design-related advantages. Laser Beam Powder Bed Fusion (LB-PBF) process is one of the metal AM processes that is extensively investigated for manufacturing commercial parts. Like the other AM processes, LB-PBF process inherently involves complex physics that often results in anisotropic and/or location specific microstructures, which are different from the conventional manufacturing routes for the same alloy [1]. While the advantages of using AM are obvious, the process-microstructure-property relationship must be well understood before utilizing AM components in critical applications that involve fatigue loading.

Alloy 718 is a precipitation strengthened iron-nickel-based superalloy. The primary strengthening precipitate is γ″, while $\gamma^{\prime }$ precipitates also contribute to its strength. δ phase in the alloy forms at the expense of γ″ and is often precipitated in a controlled manner for its beneficial influence on grain refinement and notch sensitivity. The formation of other phases such as Laves, niobium carbide (NbC) and titanium nitride (TiN) in the material is dependent on the processing route [2,3]. Alloy 718 has gained a workhorse superalloy status due to its excellent mechanical properties and corrosion resistance at a wide range of temperatures, and its processability [4]. Consequently, it has become the obvious choice to investigate the applicability of metal AM processes to superalloys. As with other metal AM processes, Alloy 718 is the most investigated superalloy with LB-PBF processing.

While significant amount of work has been done towards understanding the process-microstructure-static properties relationship for LB-PBF Alloy 718, the research on understanding its fatigue behaviour is scarce. Apart from using rotating bending and bending fatigue tests, which are discouraged by the Metallic Materials Properties Database Development and Standardization (MMPDS) for the purpose of design and analysis of structures in aerospace systems [5], fatigue studies on LB-PBF Alloy 718 have been focused on high cycle fatigue (HCF) performance evaluating the influence of defects [6,7], geometrical notches [7,8], surface roughness characteristics due to part orientation [7,9,10,11,12], and texture [13]. Furthermore, only a few investigations on low cycle fatigue (LCF) behaviour of LB-PBF Alloy 718 exist [14,15,16,17,18]. In these studies, neither the anisotropy in fatigue behaviour has been characterized thoroughly nor a method to handle this anisotropy has been proposed. Moreover, influence of the Laves phase in LB-PBF Alloy 718 on the fatigue behaviour has not been thoroughly investigated. In cast Alloy 718, Laves phase has been reported to have a detrimental effect on crack initiation and propagation behaviour [19].

A thorough understanding of the fatigue behaviour of LB-PBF Alloy 718 is required before it can replace conventionally manufactured Alloy 718 parts, especially in critical applications. Therefore, the aim of this work is to investigate the anisotropic LCF behaviour of LB-PBF Alloy 718 and establish the microstructure-fatigue behaviour relationship and propose a suitable method to handle the anisotropic strain-life relationship. For this purpose, LB-PBF Alloy 718 has been tested in three different orientations to characterize the anisotropy in fatigue behaviour. Furthermore, the material has been tested in as-built condition and after two different post-treatments to understand the effect of the different phases in the material.

## 2. Materials and Methods

### 2.1. LB-PBF Manufacturing

**Figure 1:**
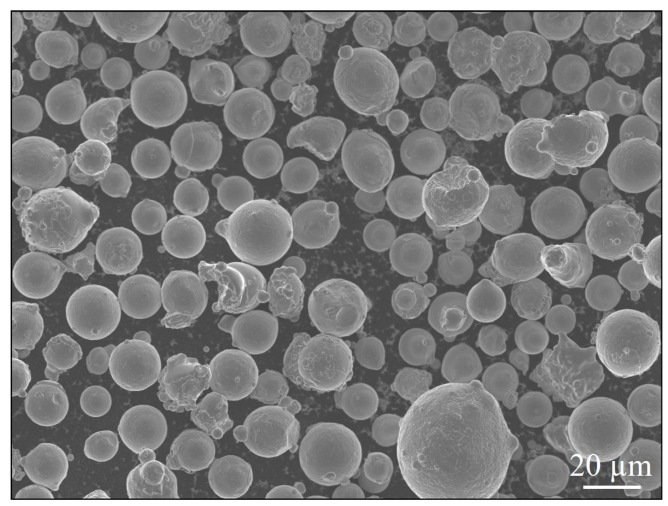
SEM image of the virgin powder.

All the specimen blanks were manufactured using an SLM 280 machine that had a twin 400 W laser configuration. The standard stripes theme v2.0 for building with Alloy 718 powder was utilized. The layer thickness, hatch spacing, hatch rotation and build plate heating were 30 μm, 120 μm, 67° and 200 °C, respectively. The hatch region was processed with a laser power of 300 W and scanning speed 1.3 m/s. Each part was built with two contour scans that were processed with a laser power of 150 W and scanning speed of 0.45 m/s. Gas atomized (GA) Alloy 718 powder that was provided by Höganäs AB having a size fraction of 15 to 45 μm and chemical composition as listed in Table 1 was utilized to manufacture the specimens. An SEM image of the virgin powder is shown in Figure 1.

**Figure 2:**
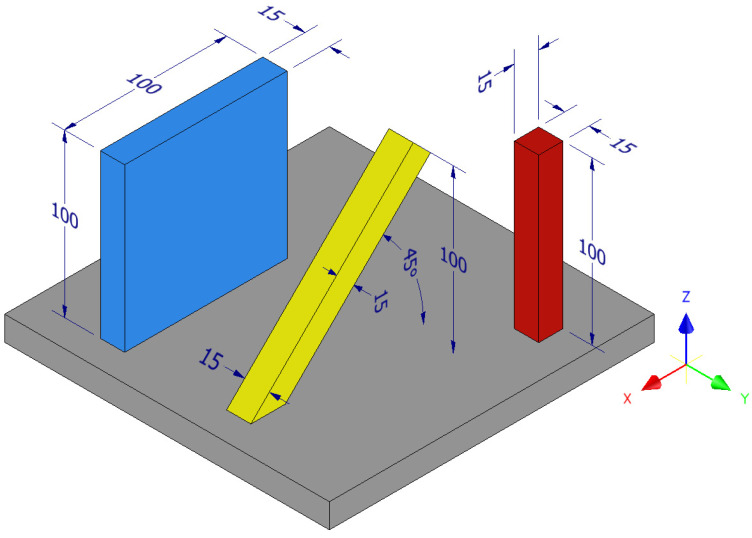
Geometry (in mm) and orientation of the specimens. Note: Z is the build direction.

To evaluate the anisotropy in the fatigue behaviour, specimen blanks were manufactured in three different orientations, namely parallel to the build direction (henceforth referred to as parallel specimens), 45° to the build direction (henceforth referred to as diagonal specimens), and transverse to the build direction (henceforth referred to as transverse specimens). Dimensions and orientation of the different specimen blanks are shown in schematic Figure 2. Each of the parallel (red in Figure 2) and diagonal (yellow in Figure 2) specimens were built as separate prismatic bars. The transverse specimens were extracted from cuboidal blocks (blue block in Figure 2).

### 2.2. Post-Treatment

The specimen blanks were sectioned off from the build platform without any stress relief treatment. The specimen blanks in all the three orientations were sorted into three groups by randomized selection. One group was utilized in the as-built (AB) condition, another was subjected to direct ageing (DA), and the last group was subjected to hot isostatic pressing (HIP) followed by solution treatment and ageing (HSA). The specifics of each of the conditions are presented in Table 2.

### 2.3. Fatigue Testing

**Figure 3:**
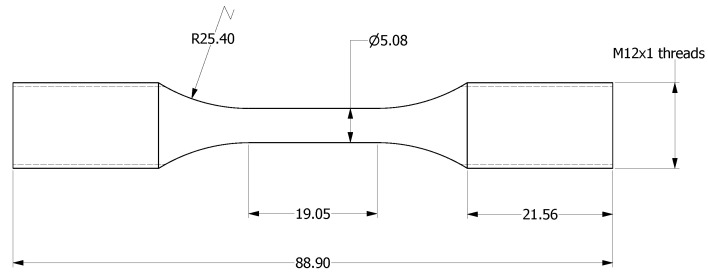
LCF specimen geometry (in mm).

Uniaxial push-pull fatigue tests were performed at room temperature under controlled amplitudes of total strain, in accordance with ASTM E606/E606M [20], using an Instron 8802 servo-hydraulic machine with 8802MT controller and LCF3 software. LCF specimens with dimensions as shown in Figure 3 were extracted, by machining, from the corresponding specimen blanks for each of the three orientations. The LCF specimens were polished to $R_{a}$ 0.2 μm. An Instron 2620-602 clip on extensometer was used to measure the strains in the gauge section over a 12.5 mm range. For each of the post-treatment and orientation combinations, at least six specimens were tested between strain amplitudes of 0.3% and 0.875% in accordance with ASTM E739 [21]. The straining cycle followed a symmetric triangular waveform at a constant frequency of 0.5 Hz. If the measured plastic strain after 43,200 cycles was less than 0.01%, the testing was switched to load-controlled cycling at 5 Hz. A 20% drop in the peak load from that of the stabilized hysteresis loop was used as the failure criterion for the test, after which the specimens were broken by applying a tensile load to reveal the fracture surfaces.

### 2.4. Material Characterization

Samples for metallographic investigation were cut, mounted, ground and polished using standard metallographic practices to observe the microstructure. The types of defects present, and their distribution were analyzed in the as-polished condition using a Zeiss AX10 light optical microscope (LOM). Image analysis technique, in accordance with ASTM E1245 [22], using the open source software Fiji [23] was performed to determine the volume fraction of the defects present.

To characterize the texture and grain size, a Zeiss Gemini 450 field emission gun (FEG) scanning electron microscope (SEM) fitted with an Oxford Symmetry Electron Back scattering Diffraction (EBSD) detector was utilized. Samples for texture analysis were polished with a 0.02 μm silica suspension using a vibropolishing machine. EBSD analysis was performed on sections both parallel and perpendicular to the build direction. Texture analysis was performed using Aztec Crystal v1.1 software, while grain size analysis was performed, by line intercept method, using ATEX software [24] in accordance with ASTM E2627 [25] and ASTM E1382 [26].

Electrolytic etching was performed with oxalic acid at 3 V for 5–10 s or with 50:50 Kalling’s reagent:ethanol solution at 2 V for 3–5 s to reveal the microstructure. Microstructure characterization was carried out using the Zeiss AX10 LOM and the Zeiss Gemini 450 FEG SEM fitted with an Oxford ULTIM MAX 100 mm${}^{2}$ X-ray energy-dispersive spectroscopy (EDS) detector. Chemical analysis was performed using Aztec v4.2 software.

To observe the dislocation structures using Transmission Electron Microscopy (TEM), thin strips were cut along the loading direction. The strips were ground to a thickness of 100 μm and discs, 3 mm in diameter, were punched out. The discs were subsequently thinned down further using electropolishing. Electron transparency in the discs was obtained using a twinjet polishing machine (Tenupol-5) operating at −30 °C and 20.5 V with 10% perchloric acid in methanol as electrolyte. FEI transmission electron microscope, Tecnai G2 with double-tilt sample holder was used to enable orienting the samples to two-beam conditions during dislocation analysis.

Fractography was performed using an Olympus SZX9 stereomicroscope and a Zeiss EVO 50 SEM.

## 3. Results and Discussion

### 3.1. Microstructure

The test specimens were extracted, by machining, from the hatch region. Therefore, only the microstructure from the hatch region is presented. Microstructure characterization was performed on several metallographic samples extracted from all the three types of specimen blanks shown in Figure 2. The microstructure was identical in the three types of specimen blanks (for brevity, only the representative microstructure is presented here), and is as follows.

#### 3.1.1. Grain Morphology and Texture

**Figure 4:**
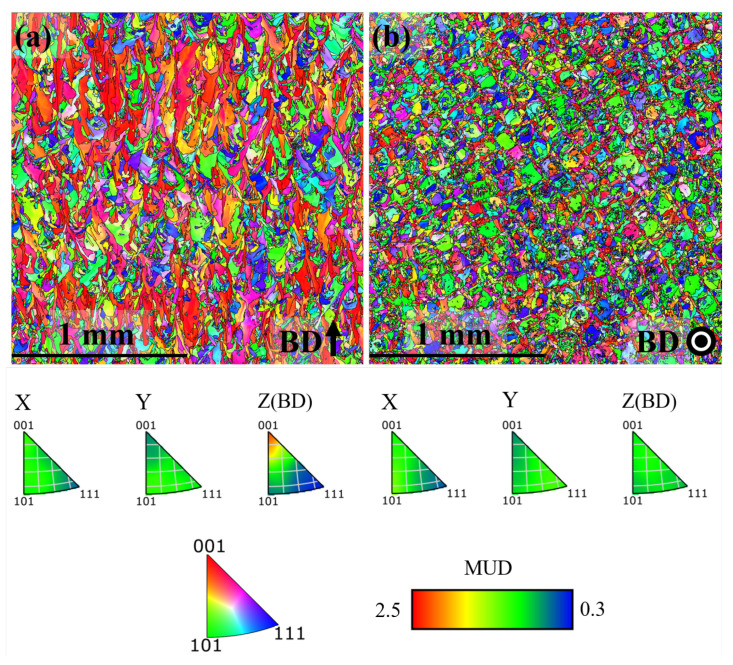
IPF map in the AB condition (**a**) x-z plane (**b**) x-y plane.

**Figure 5:**
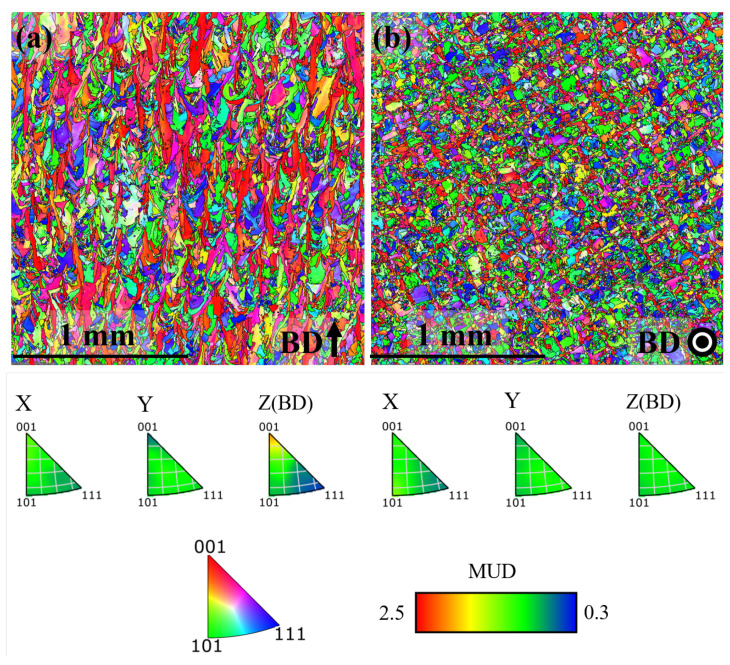
IPF map in the DA condition (**a**) x-z plane (**b**) x-y plane.

**Figure 6:**
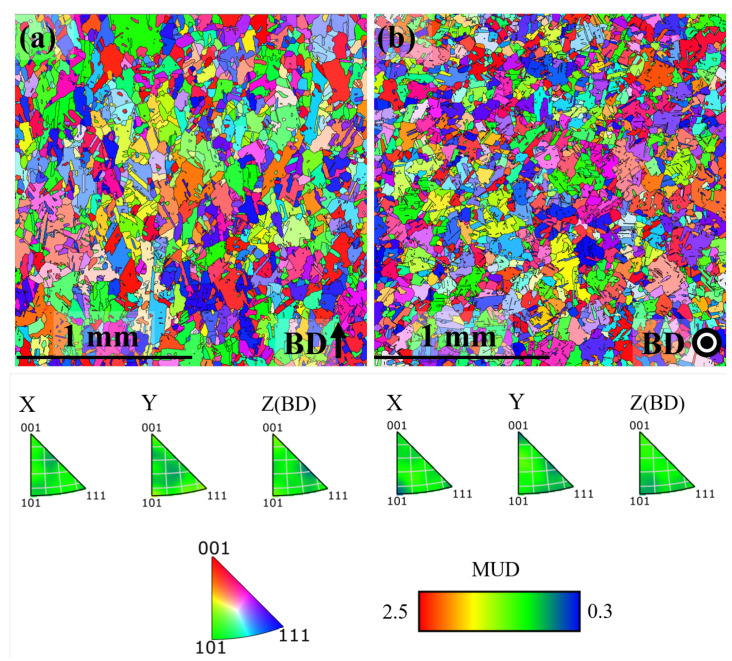
IPF map in the HSA condition (**a**) x-z plane (**b**) x-y plane.

**Figure 7:**
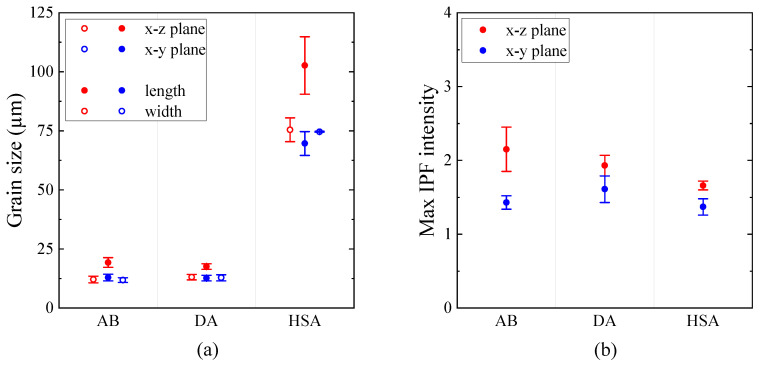
(**a**) Grain size (**b**) IPF texture intensity in different post-treatment conditions.

EBSD inverse pole figure (IPF) maps of the microstructure in x-z plane and x-y plane for the AB, DA and HSA conditions are shown in Figure 4, Figure 5 and Figure 6, respectively. Grain size (length along Z and width along X in the x-z plane, length along Y and width along X in the x-y plane) in the different post-treatment conditions is shown in Figure 7a; the corresponding maximum IPF texture intensity is presented in Figure 7b.

In the AB condition, as in Figure 4a, grains were elongated in the build direction with an aspect ratio of ∼1.5. The grains were generally pointing upward, but not aligned with the build direction. As shown in Figure 4b, the grains were equiaxed perpendicular to the build direction. Furthermore, a weak <100> texture along the build direction existed with an intensity of ∼2.2 MUD (multiples of uniform density). These observations are in agreement with the as-built microstructural features commonly reported for LB-PBF Alloy 718 [27,28,29]. In the DA condition, as shown in Figure 5, the microstructure in terms of the grain morphology and texture was identical to that of the AB condition, which is in agreement with the published literature [27,28]. In the HSA condition, as shown in Figure 6 and Figure 7a, grains were ∼4 times larger than in the AB and DA conditions. The texture intensity, as shown in Figure 7b, was lower than both the AB and DA conditions. Furthermore, the grains were elongated along the build direction with an aspect ratio of ∼1.5 and were equiaxed perpendicular to the build direction. The maximum IPF texture intensity was relatively higher parallel to the build direction than perpendicular to it. Both these facts indicate that the material has undergone recovery and grain growth, and not the commonly reported recrystallization and grain growth [28,30].

#### 3.1.2. Sub-Grain Morphology and Secondary Phases

**Figure 8:**
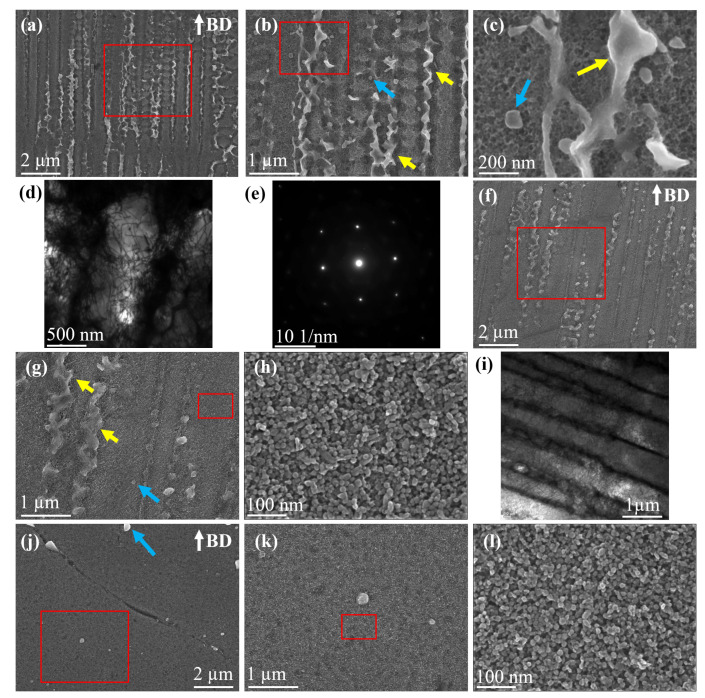
(**a**–**e**) AB condition. (**a**–**c**) SEM images at different magnifications showing the cellular morphology, Laves phase and NbC. (**d**) TEM image showing highly dense dislocation network at the cell boundaries. (**e**) SAED patter showing absence of superlattice reflections of the strengthening precipitates. (**f**–**i**) DA condition. (**f**–**h**) SEM images at different magnifications showing the cellular morphology, Laves phase, NbC and strengthening precipitates. (**i**) TEM image showing highly dense dislocation network at the cell boundaries. (**j**–**l**) SEM images at different magnifications showing NbC and strengthening precipitates in the HSA condition. Note: Yellow arrows point to Laves Phase, blue arrows point to NbC.

The sub-grain morphology and secondary phases in the AB, DA and HSA conditions are shown in Figure 8. In the AB condition, as shown in Figure 8a–e, the typically reported segregated cellular morphology was found. Highly dense network of dislocations, that were inherited from the rapid solidification during LB-PBF processing, were present at the cell boundaries, as shown in the TEM image in Figure 8d. Laves phase and NbC were found at the intercellular sites. The strengthening precipitates were absent in the AB condition, as shown in the selected area electron diffraction (SAED) pattern in Figure 8e. In the DA condition, as shown in Figure 8f–i, the cellular morphology and dense dislocation network at the cell boundary were present like the AB condition. The temperature during the ageing treatment was insufficient to promote complete annealing/recovery and subsequent grain growth in the material. The Laves phase and NbC were present at the intercellular sites; furthermore, the γ″ and $\gamma^{\prime }$ strengthening precipitates were present in the matrix following to the two step ageing treatment. In the HSA condition, as shown in Figure 8j–l, the cellular substructure and Laves phase were absent. NbC, relatively larger in size compared to the AB and DA condition, were present along with the γ″ and $\gamma^{\prime }$ precipitates. The high temperature during the HIP stage of HSA is, normally, sufficient to dissolve the Laves phase, to anneal the dislocation structure and to trigger recovery and grain growth. The dissolution of Laves phase consequently leads to coarsening of NbC particles.

#### 3.1.3. Defects

**Figure 9:**
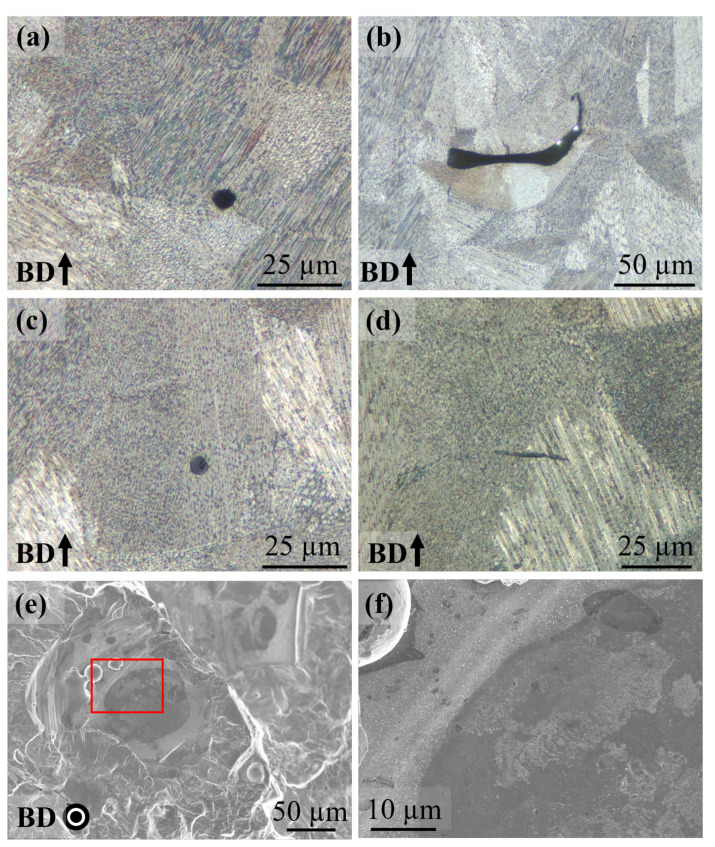
Defects in AB condition. (**a**) Porosity (**b**) LoF (**c**,**d**) inclusions (**e**) LoF from a fracture surface (**f**) magnified image of area indicated in (**e**).

In the AB condition, defects such as porosity and lack of fusion (LoF) were present (Figure 9a,b). Besides these defects, oxide inclusions rich in Al and Ti were also present (Figure 9c,d). The defects and inclusions were randomly distributed in the hatch region. Several LoF defects had oxide particles present at the surface; this was identified from the LoF defects in the fracture surfaces as shown in Figure 9e,f. In the DA condition, the volume fraction and distribution of the defects and inclusions were identical to that of the AB condition (for brevity these are not shown). In the HSA condition, a few remnant pores were present, but, the LoF defects were absent. Furthermore, the inclusions such as the ones in Figure 9d were present at a higher frequency than the other two conditions; these were found at the same frequency and distribution as the LoF defects, which could be due to closure of the LoF defects that have inclusions at the surface. The presence of LoF defects and inclusions in the material used in this study are attributed to issues with the powder recoating and Ar gas flow in the machine. The issues with the recoating and gas flow were identified during the root-cause analysis performed after the completion of the fatigue testing.

### 3.2. Cyclic Stress Evolution

#### 3.2.1. First Cycle Data

Young’s modulus in the different material conditions was obtained from the first loading cycle and is presented in Table 3. The modulus was anisotropic in the AB and DA conditions, which is due to the <100> fiber texture in the material. Correspondingly, the modulus was the lowest in the parallel direction (which is the building direction) that had <100> texture. As a consequence of <100> fiber texture along the parallel direction, the main texture component along the diagonal direction is of <101> fiber type; therefore, the modulus was the highest in the diagonal direction. In the transverse direction, the relatively homogeneous texture resulted in a moderate modulus. The relatively weaker/nearly uniform texture in the HSA condition, compared to the AB and DA conditions, resulted in an isotropic Young’s modulus.

The difference in yield strength between the different post-treatments could be assessed from the first cycle stress range of specimens that exhibit significant cyclic plastic strains. The first cycle stress range from specimens subjected to strain amplitude of 0.75% are presented in Table 4. The AB condition required the lowest stress range and is therefore the softest, which is due to the absence of strengthening precipitates. Among the DA and HSA conditions that contain strengthening precipitates, the DA condition that was composed of a finer grain size (refer Figure 7b) required a higher stress range. The relatively higher strength in the DA condition could be explained qualitatively by the Hall-Petch relationship. In the AB and DA conditions, stress range was the highest in the diagonal orientation, the lowest in the parallel orientation and moderate in the transverse orientation following the trend in the anisotropic Young’s modulus. Furthermore, stress range in the HSA condition was equivalent in the three orientations indicating an isotropic behaviour in strength.

#### 3.2.2. Hysteresis Loops

**Figure 10:**
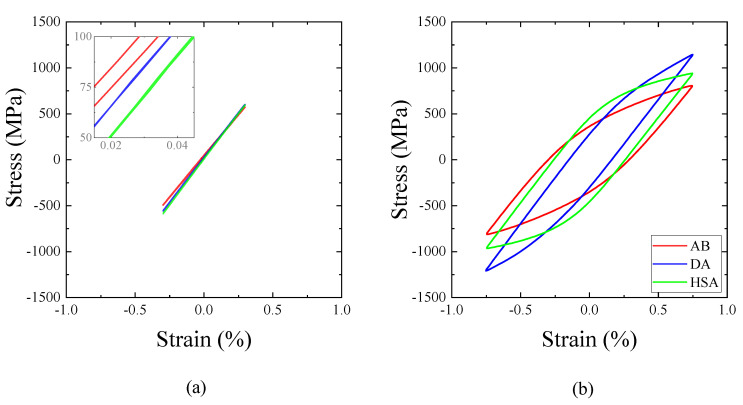
Hysteresis of transverse specimens in different post-treatments at (**a**) $\epsilon_{a}$ = 0.3% and (**b**) $\epsilon_{a}$ = 0.75%.

Mid-life hysteresis loops of the transverse specimens at strain amplitude of 0.3% and 0.75% are presented in Figure 10; the other two orientations are not shown for brevity, but the behaviour was identical. At the lowest strain amplitude of 0.3%, the cyclic behaviour was purely elastic in the DA and HSA conditions; however, the AB condition exhibited a small cyclic plastic strain as shown in the inset in Figure 10a. As discussed earlier, the strength is lower in the AB condition due to which the material undergoes cyclic plasticity even at 0.3% strain amplitude. At higher strain amplitudes, for example at 0.75%, all the material conditions exhibited cyclic plasticity as shown in Figure 10b. The magnitude of cyclic plastic strain was the highest in the AB condition, moderate in the HSA condition, and the lowest in the DA condition. This observed trend in the cyclic plastic strain, as expected, has an inverse relationship with the yield strength in the different conditions.

#### 3.2.3. Cycle Dependence of Stress Evolution

**Figure 11:**
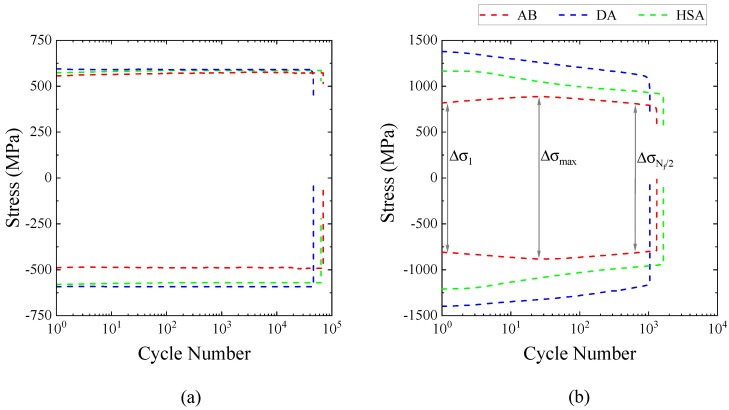
Stress evolution of transverse specimens in different post-treatments at (**a**) $\epsilon_{a}$ = 0.3% and (**b**) $\epsilon_{a}$ = 0.75%.

**Figure 12:**
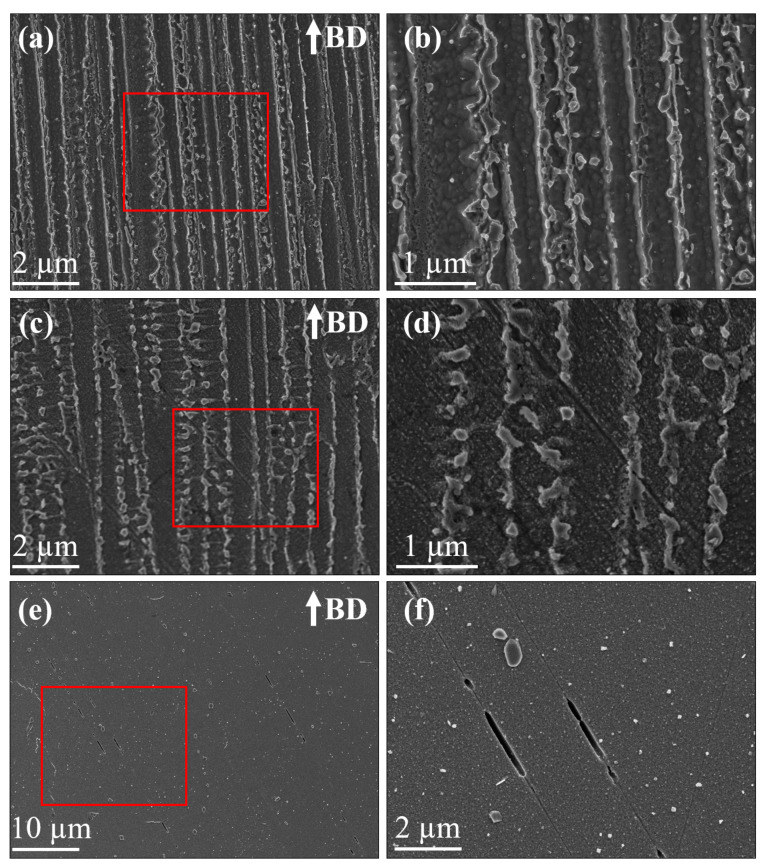
SEM images of the microstructure post-deformation. (**a**,**b**) AB condition having no planar slip bands and un-sheared Laves phase particles. (**c**,**d**) DA condition showing planar slip bands and some sheared Laves phase particles. (**e**,**f**) HSA condition showing planar shear bands.

At strain amplitudes that caused only an elastic cyclic response, a pronounced level of cyclic saturation was observed until failure irrespective of the post-treatment condition, as shown in Figure 11a. At higher strain amplitudes that induce significant cyclic plasticity, a difference in the cyclic stress evolution behaviour was observed depending on the post-treatment condition, as shown in Figure 11b. The DA and HSA conditions exhibited a continuous cycle-dependent softening behaviour until failure, whereas the AB condition exhibited an initial cycle-dependent hardening followed by softening until failure. The softening behaviour in the DA and HSA conditions could be explained by the precipitate shearing mechanism under cyclic loading [31,32]. Due to the precipitate shearing, planar slip bands (as shown in Figure 12d,f) form that act as easy path for further dislocation movement. The formation of planar slip bands is responsible for the continuous softening response [32]. In the AB condition, in the absence of strengthening precipitates, the deformation is homogeneous and is not localized in planar slip bands (as shown in Figure 12b). Such homogeneous deformation while the precipitate shearing mechanism is inactive is well known [33]. The deformation-induced dislocations are homogeneously distributed in the matrix and their interaction causes the initial hardening. Upon continued cyclic deformation, rearrangement of these dislocations leads to softening. This observed difference in the deformation behaviour and cyclic stress evolution, influenced by the strengthening precipitates, has been reported for both wrought [34] and LB-PBF Alloy 718 [16] as well. This behaviour for the transverse specimens, for brevity, is presented in Figure 11b and for all the orientations, at a few selected strain amplitudes, in Table 5. The ratio of maximum stress range and first cycle stress range indicates the magnitude of hardening, while the ratio of maximum stress range and mid-life stress range indicates the magnitude of softening. In general, the hardening and softening increased with an increase in applied strain amplitude.

#### 3.2.4. Asymmetric Stress Response

Furthermore, from the cyclic stress evolution, stress ratio ($R_{\sigma }$) of the first and mid-life cycles were determined to evaluate symmetry/asymmetry of the stress response. The $R_{\sigma }$ was calculated based on true stress, instead of engineering stress, to account for the difference in cross sectional area between tension and compression segments of the loading cycle. The $R_{\sigma }$ for the different material conditions at a few selected strain amplitudes is presented in Table 6. In general, the AB condition exhibited significant asymmetry, while the DA and HSA conditions exhibited only minor asymmetry. The minor asymmetry in the DA and HSA conditions could be related to the intrinsic tension-compression asymmetry generally exhibited by the material [35]. In the AB condition, the observed asymmetry was orientation dependent i.e., in the parallel and diagonal orientations, the asymmetry was in the compression direction, whereas in the transverse specimens the asymmetry was in the tensile direction. Therefore, in the AB condition, other factors could contribute to the observed asymmetry besides the intrinsic tension-compression asymmetry.

**Figure A1:**
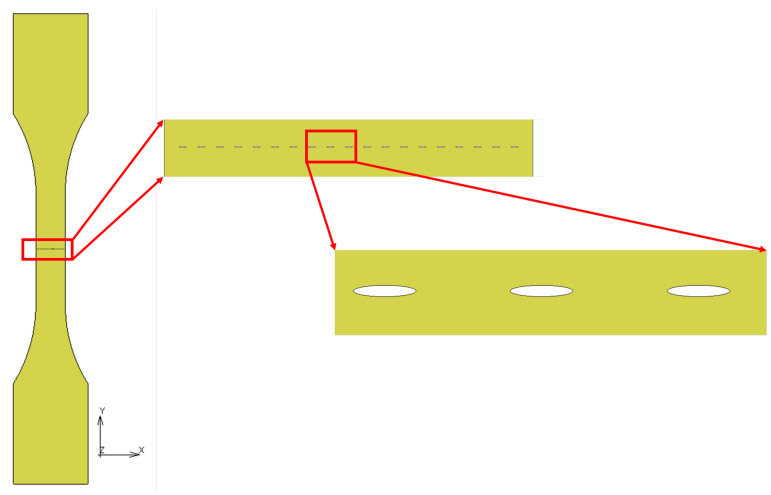
FE model.

**Figure A2:**
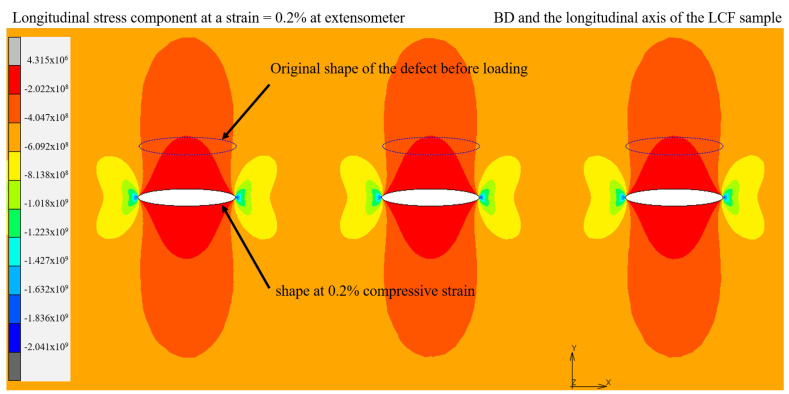
FE simulation result.

In the presence of LoF defects, the net load-bearing cross sectional area could be different between tension and compression segments of the loading cycle if the faces of the LoF defects contact each other during compression. To assess this hypothesis, simple 2D finite element (FE) analysis was performed by introducing elliptical defects with the dimensions identified from optical microscopy (shown in Figure A1 in Appendix A). Defect closure i.e., contact between the faces of the defects did not occur even when multiple defects were assumed to be at the same line and a compressive displacement corresponding to the highest applied strain amplitude (shown in Figure A2 in Appendix A). Furthermore, in the DA condition, despite the similar distribution of defects, the asymmetry was insignificant. Therefore, it was concluded that the LoF defects had minimal influence towards the asymmetric stress evolution in the AB condition. Since the actual defects could have an acute radius of curvature than an ellipse utilized in the FE model, the influence of defects could not be completely discounted. However, the influence would not be significant. LB-PBF manufactured parts, in the AB condition, have been reported to have significant magnitudes of residual stress [36,37]. Besides, the asymmetry was higher at lower strain amplitudes and vice versa. With increased cyclic plasticity at higher strain amplitudes, the asymmetry diminished due to cycle-dependent relaxation as shown in Table 6. So, the residual stress could contribute towards the asymmetry in the AB condition. Furthermore, the lower asymmetry in the DA condition that had similar defect distribution as the AB condition supports the hypotheses that residual stress could have a significant contribution, while the defects have a negligible influence. Further systematic investigation of the influence of residual stress on the cyclic stress evolution behaviour is needed to characterize this behaviour.

### 3.3. Strain Amplitude vs. Fatigue Life

**Figure 13:**
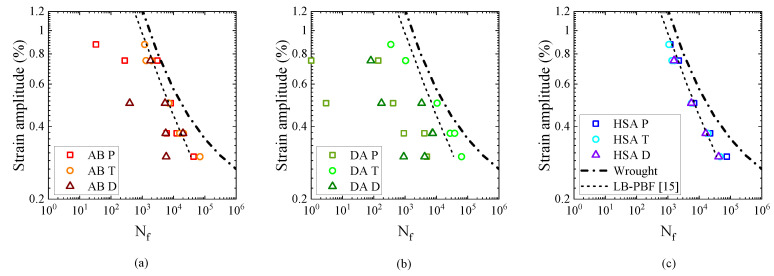
Strain vs. life plots for the different post-treatments. (**a**) AB condition. (**b**) DA condition. (**c**) HSA condition.

**Figure 14:**
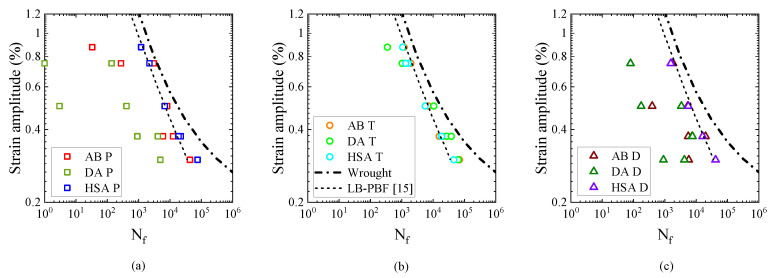
Strain vs. life plots for the different orientations. (**a**) Parallel orientation. (**b**) Transverse orientation. (**c**) Diagonal orientation.

**Figure 15:**
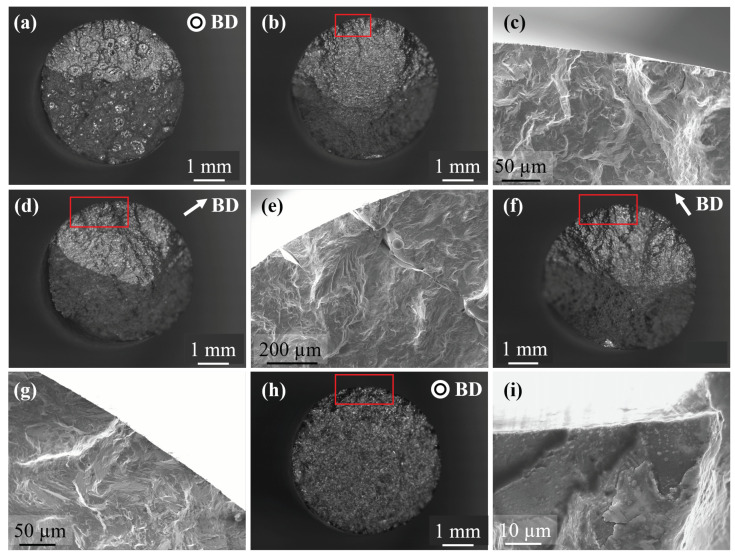
SEM fractographs. (**a**) AB parallel specimen with numerous LoF defects. (**b**,**c**) AB parallel specimen without LoF defects in the fracture surface and crack initiation from the surface. (**d**,**e**) AB transverse specimen with crack initiation from LoF defect at the surface. (**f**,**g**) AB transverse specimen without LoF defects in the fracture surface and crack initiation from the surface. (**h**,**i**) HSA diagonal specimen with tiny inclusions at the crack initiation site.

Strain amplitude vs. fatigue life plots for the different post-treatment conditions is presented in Figure 13. The same data is re-plotted for the different orientations as in Figure 14. In both the figures, data from literature for wrought Alloy 718 [38] as well as LB-PBF Alloy 718 [15] (subjected to stress relief + HSA) have been plotted. In the parallel and diagonal orientations, in both the AB and DA conditions, a significant scatter in fatigue life was observed; however, in the transverse orientation, in both the conditions, the scatter was lower. The higher scatter in the parallel and diagonal orientations is due to the presence of numerous LoF defects that act as crack initiation sites (Figure 15a–c). Similar defect-induced scatter in strain-controlled fatigue testing of LB-PBF Alloy 718 have been reported [14,15]. In the parallel specimens, LoF defects were oriented normal to the loading direction and in the diagonal specimens, LoF defects were oriented at 45° to the loading direction. Therefore, in these two orientations the LoF defects cause significant stress concentration and eventually initiate cracks at the sharp edges. In the transverse specimens, however, LoF defects are oriented parallel to the loading direction and are not as detrimental as the other two cases. Though the presence of LoF defects is a discontinuity and causes stress concentration, cracks are not initiated since the sharp edges of the defects are aligned with the loading direction. Therefore, in the transverse specimens LoF defects cause crack initiation only if located at the surface (Figure 15d,e). Such an anisotropic fatigue behaviour in metal AM that is related to the orientation of LoF defects with respect to the loading direction is well known [39]. In general, when LoF defects are oriented along the loading direction the fatigue performance is better, which is also observed in the present study. The fatigue life is relatively lower in the DA condition than the AB condition, as shown in Figure 14a,c. The relatively higher strength in the DA condition leads to a higher notch sensitivity. Consequently, the crack initiation is relatively easier, which leads to lower life. In the HSA treated condition, all three orientations had equivalent fatigue performance as shown in Figure 13c. Thus, the HIP treatment that eliminated most of the defects nullified the defect-induced anisotropy in the fatigue performance. The fatigue performance of the HSA treated material is equivalent to the published LB-PBF 718 data [15], but is worse than the wrought data due to the inclusions associated with crack initiation (Figure 15h,i).

**Figure 16:**
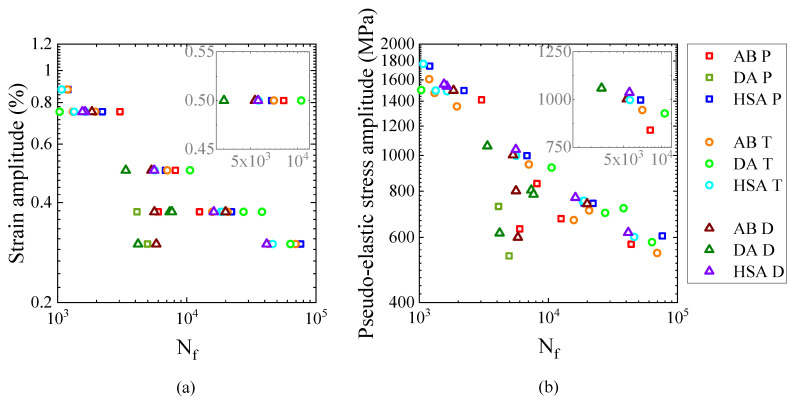
(**a**) Strain vs. life plots (**b**) Pseudo-elastic stress vs. life plots.

Furthermore, in the transverse orientation, as in Figure 14b, the fatigue life in the AB and DA conditions was equivalent to that of the HSA treated specimens, which indicates that the orientation of LoF defects with respect to the loading direction has the largest impact on the fatigue performance. In cases when the fatigue life was poorer than the HSA condition, the fatigue crack had initiated at a defect located at the surface. When the fatigue crack initiation was not associated with a defect, the fatigue life in the AB and DA conditions was better than the HSA condition. Similar inferences could be drawn from the other two orientations as well, which is shown in Figure 16a. The Figure 16a shows all the data from Figure 13 (in $10^{3}$ and $10^{5}$ life range) to reveal that the fatigue life in the AB and DA conditions, sometimes, exceed that of the HSA condition. Similar reduction in fatigue performance of the HSA treated LB-PBF Alloy 718 has been reported, but, the reasons were not explored thoroughly [16]. The lower fatigue life in the HSA condition is somewhat counter intuitive. The HIP treatment healed most of the defects and reduced the defect-induced anisotropy; therefore, a higher fatigue life in the HSA condition would be expected. Furthermore, since Laves phase is present in both the AB and DA conditions, lower fatigue performance would be expected as Laves phase has been reported to be preferential site for crack initiation and crack propagation in cast Alloy 718 [19]. However, the size fraction of Laves phase in LB-PBF Alloy 718 is smaller than that of cast material, which could explain the absence of detrimental effect of Laves phase in the present case.

In strain-controlled fatigue testing, the stress response is proportional to the Young’s modulus i.e., to achieve a given total strain, the material that has higher Young’s modulus would require higher stress and vice versa. Therefore, the material that has a higher modulus would experience a higher stress and consequently a higher critical resolved shear stress, which results in higher deformation and eventually lower fatigue life. As discussed earlier, the Young’s modulus was different between the different orientations and the post-treatment conditions (refer Table 3). The equivalent modulus of the three orientations in the HSA condition rendered equivalent fatigue life, and only the material with a lower modulus than the HSA condition have better fatigue life, both of which are in accordance with the hypothesis. Similar elasticity-induced anisotropic fatigue behaviour has been reported for EB-PBF Alloy 718 [40]. It has also been shown that the pseudo-elastic stress amplitude vs. fatigue life approach could be used to handle the anisotropy observed in the strain-life relationship [40]. The pseudo-elastic stress amplitude is estimated from the applied strain amplitude ($\epsilon_{a}$) and the Young’s modulus (*E*) as $\sigma_{pseudo-elastic}=\epsilon_{a}.E$. Pseudo-elastic stress amplitude vs. fatigue life plot is shown in Figure 16b. In this plot, the data points collapse to a linear relationship with a relatively lower scatter, which is highlighted in the insets in Figure 16a,b. The data points that deviate from the linear relationship are related to crack initiations from large LoF defects. Therefore, the texture-induced elasticity-related anisotropy of the fatigue behaviour, under controlled amplitudes of strain, in metal AM could be modelled using the pseudo-elastic stress vs. fatigue life approach.

## 4. Conclusions

In this study, fatigue behaviour of LB-PBF Alloy 718 has been investigated under controlled amplitudes of strain. LB-PBF Alloy 718 was tested in three post-treatment conditions (AB, DA and HSA) and three orientations (parallel, transverse and diagonal to the build direction). The specimen were extracted by machining and the surfaces were polished to $R_{a}$ 0.2 μm. The findings are summarized as follows.

1. The presence of strengthening precipitates resulted in the formation of planar slip bands that led to cycle-dependent softening, while their absence resulted in homogeneous deformation that led to initial hardening before the onset of softening.
2. LoF defects and inclusions have a detrimental effect on fatigue life. The LoF defects in the AB and DA conditions resulted in a significant scatter in fatigue life. The inclusions were not affected by the HIP treatment and were responsible for lower fatigue life of the HSA treated material than the MMPDS wrought data.
3. The presence of Laves phase in the AB and DA conditions did not have a detrimental effect on fatigue life.
4. Besides the orientation of LoF defects relative to the loading direction, the anisotropy in Young’s modulus in the AB and DA conditions contributed to the anisotropy in the fatigue life.
5. The lower fatigue life in the HSA condition than the AB and DA conditions (when the crack initiation is unrelated to defects) could be explained by the difference in Young’s modulus between the conditions.
6. The anisotropy in strain-life behaviour could be treated using the pseudo-elastic stress vs. fatigue life approach.

## Figures and Tables

**Table 1:** Nominal chemical composition of GA Alloy 718 powder.

**Element**	Ni	Fe	Cr	Nb	Mo	Ti	Al	C
**wt%**	53.00	bal	18.80	5.42	3.10	1.10	0.60	0.04

**Table 2:** Post-treatment conditions.

Condition	HIP	Solution Treatment	Ageing
**AB**	none	none	none
**DA**	none	none	718 °C/8 h/FC to 621 °C + 621 °C/8 h/AC
**HSA**	1160 °C/4 h/100 MPa/URC	1065 °C/1 h/AC	718 °C/8 h/FC to 621 °C + 621 °C/8 h/AC

**URC**: Uniform Rapid Cooling; **FC**: Furnace Cooling @ 55 °C/h; **AC**: Air cooling.

**Table 3:** Young’s modulus (GPa) in different orientations and post-treatments.

Orientation	AB	DA	HSA
**Parallel**	176 ± 12	176 ± 13	199 ± 1
**Transverse**	187 ± 6	190 ± 6	200 ± 1
**Diagonal**	203 ± 5	212 ± 4	206 ± 2

**Table 4:** Stress range (MPa) at $\epsilon_{a}$ = 0.75% in different orientations and post-treatments.

Orientation	AB	DA	HSA
**Parallel**	1456	2617	2359
**Transverse**	1534	2776	2361
**Diagonal**	1622	2910	2386

**Table 5:** Cyclic stress evolution behaviour of different material conditions.

Material Condition	$\epsilon_{a}=0.375\%$		$\epsilon_{a}=0.5\%$		$\epsilon_{a}=0.75\%$	
	$\Delta \sigma_{\max}/\Delta \sigma_{1}$	$\Delta \sigma_{\max}/\Delta \sigma_{N_{f}/2}$	$\Delta \sigma_{\max}/\Delta \sigma_{1}$	$\Delta \sigma_{\max}/\Delta \sigma_{N_{f}/2}$	$\Delta \sigma_{\max}/\Delta \sigma_{1}$	$\Delta \sigma_{\max}/\Delta \sigma_{N_{f}/2}$
**AB Parallel**	1.03	1.00	1.06	1.03	1.09	1.11
**AB Transverse**	1.05	1.01	1.07	1.06	1.09	1.09
**AB Diagonal**	1.02	1.02	1.08	1.07	1.09	1.11
**DA Parallel**	1.01	1.00	1.00	1.00	1.00	1.11
**DA Transverse**	1.01	1.00	1.01	1.01	1.00	1.18
**DA Diagonal**	1.00	1.01	1.01	1.01	1.00	1.11
**HSA Parallel**	1.01	1.01	1.00	1.14	1.00	1.26
**HSA Transverse**	1.00	1.02	1.00	1.11	1.00	1.25
**HSA Diagonal**	1.01	1.02	1.00	1.15	1.00	1.25

**Table 6:** Stress ratio ($R_{\sigma }$) evolution with respect to different applied strain amplitudes.

Material Condition	$\epsilon_{a}=0.375\%$		$\epsilon_{a}=0.5\%$		$\epsilon_{a}=0.75\%$	
	1*^st^*	$N_{f}/2$	1*^st^*	$N_{f}/2$	1*^st^*	$N_{f}/2$
**AB Parallel**	−1.31	−1.32	−1.27	−1.23	−1.13	−1.05
**AB Transverse**	−0.85	−0.87	−0.91	−0.95	−0.95	−0.99
**AB Diagonal**	−1.08	−1.08	−1.06	−1.04	−1.03	−1.01
**DA Parallel**	−1.02	−0.98	−1.02	−1.02	−1.02	−1.03
**DA Transverse**	−1.01	−1.02	−1.01	−1.01	−0.98	−1.01
**DA Diagonal**	−1.01	−1.06	−1.02	−1.08	−1.03	−1.06
**HSA Parallel**	−0.99	−0.99	−1.01	−0.98	−1.03	−1.00
**HSA Transverse**	−1.01	−1.00	−1.01	−1.02	−1.03	−1.01
**HSA Diagonal**	−1.02	−1.02	−1.01	−1.02	−1.03	−1.02

## Abbreviations

**2D**	Two Dimensional
**AB**	As-built
**AC**	Air cooling
**AM**	Additive Manufacturing
**ASTM**	American Society for Testing and Materials
**DA**	Direct Ageing
**EBSD**	Electron Backscatter Diffraction
**FC**	Furnace Cooling
**FE**	Finite Element
**FEG**	Field Emission Gun
**GA**	Gas Atomized
**HCF**	High Cycle Fatigue
**HIP**	Hot Isostatic Pressing
**HSA**	HIP + Solution treatment + Ageing
**LB-PBF**	Laser Beam Powder Bed Fusion
**LCF**	Low Cycle Fatigue
**LoF**	Lack of Fusion
**LOM**	Light Optical Microscope
**MUD**	Multiples of Uniform Density
**NbC**	Niobium Carbide
**PBF**	Powder Bed Fusion
**SAED**	Selected Area Electron Diffraction
**SEM**	Scanning Electron Microscope
**TEM**	Transmission Electron Microscope
**TiN**	Titanium Nitride
**URC**	Uniform Rapid Cooling

## Appendix A. Finite Element Simulations

A finite element (FE) model was setup using MSC Marc (Ver. 2019) to investigate if the defects observed in the AB condition could close during the compression segment of the LCF loading cycle. To simplify the computation, a 2D plane strain modelling approach was used. 1:1 2D geometry of the specimen was created and 19 elliptical defects (major axis of 100 μm and minor axis of 18 μm) were designed along a line at the centre of the gauge section as shown in Figure A1. Defects were oriented such that the major axis was normal to the loading axis of the specimen. This was a hypothetical worst case scenario as defects were distributed more randomly and not at the same plane, as identified during fractoraphy and metallography.

The specimen was fixed at the bottom and a displacement was applied at the top to simulate the compression segment of the LCF loading cycle. Young’s modulus of 200 GPa and poison’s ratio of 0.3 were used. Plasticity data from the first cycle response, from the experiments involving significant plastic strains, was utilized. In the model, strains were extracted at a location equivalent to the extensometer location in the experiments.

A compressive displacement corresponding to the highest strain amplitude in the testing does not lead to closure of the defects i.e., the defect faces did not contact each other as shown in Figure A2.
